# Embracing robotic surgery in low- and middle-income countries: Potential benefits, challenges, and scope in the future

**DOI:** 10.1016/j.amsu.2022.104803

**Published:** 2022-11-01

**Authors:** Aashna Mehta, Jyi Cheng Ng, Wireko Andrew Awuah, Helen Huang, Jacob Kalmanovich, Aniket Agrawal, Toufik Abdul-Rahman, Mohammad Mehedi Hasan, Vladyslav Sikora, Arda Isik

**Affiliations:** a University of Debrecen-Faculty of Medicine, Debrecen, 4032, Hungary; b Faculty of Medicine and Health Sciences, University of Putra Malaysia, Serdang, Malaysia; c Sumy State University and Toufik's World Medical Association, Sumy, Ukraine; d Royal College of Surgeons in Ireland, University of Medicine and Health Science, Dublin, Ireland; e Drexel University, College of Medicine, Philadelphia, PA, USA; f Department of Pediatric Surgery, Center for Children, Kokilaben Dhirubhai Ambani Hospital and Medical Research Institute, Mumbai, India; g Department of Biochemistry and Molecular Biology, Faculty of Life Science, Mawlana Bhashani Science and Technology University, Tangail, Bangladesh; h Istanbul Medeniyet University, Department of General Surgery, Istanbul, Turkey

## Abstract

Robotic surgery has applications in many medical specialties, including urology, general surgery, and surgical oncology. In the context of a widespread resource and personnel shortage in Low- and Middle-Income Countries (LMICs), the use of robotics in surgery may help to reduce physician burnout, surgical site infections, and hospital stays. However, a lack of haptic feedback and potential socioeconomic factors such as high implementation costs and a lack of trained personnel may limit its accessibility and application. Specific improvements focused on improved financial and technical support to LMICs can help improve access and have the potential to transform the surgical experience for both surgeons and patients in LMICs. This review focuses on the evolution of robotic surgery, with an emphasis on challenges and recommendations to facilitate wider implementation and improved patient outcomes.

## Highlights

• Recent advances in robotic surgery have the potential to improve surgical services.
• In LMICs, it can reduce the risk of physician fatigue, surgical site infections and improve quality of surgical care.
• Lack of infrastructure, training, and expensive equipment pose significant barriers to robotics integration in LMICs.
• Addressing challenges and focusing on region specific improvements can help bridge the surgical gap to underserved communities.

## 1 Introduction

**Fig. 1:**
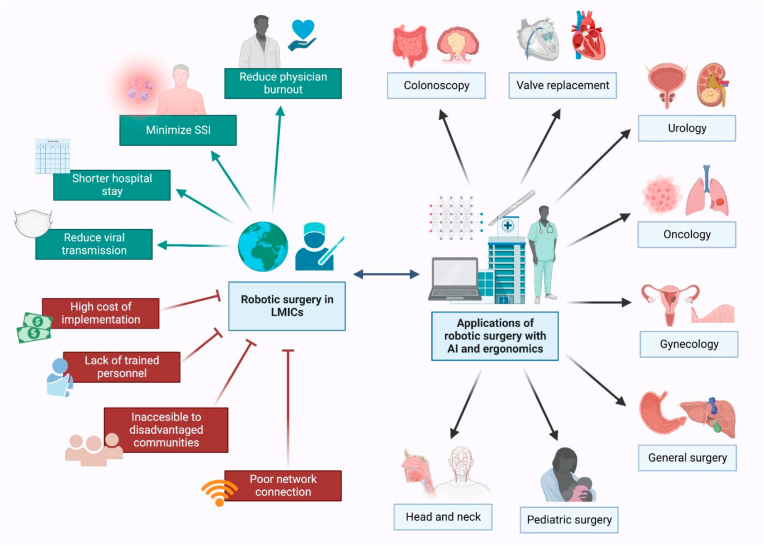
The applications of robotic surgery with AI and ergonomics in a variety of surgical sub-specialties can transform surgical care in LMICs (green), but there are major barriers that hinder its widespread implementation (red); AI: Artificial Intelligence, LMICs: Low- and middle-income countries, SSi: Surgical Site Infections (Created with biorender.com). (For interpretation of the references to color in this figure legend, the reader is referred to the Web version of this article.)

Robotic surgery, also known as robot-assisted surgery, is a revolutionary technology that has enabled more minimally invasive and precise approaches, resulting in less wound access trauma, shorter hospital stays, improved surgical visualization, greater surgical precision, and fewer postoperative wound complications [1] (see Fig. 1).

The first surgical robot, PUMA560, was introduced in 1985 to perform a CT-guided brain biopsy [2]. In 2000, the da Vinci system (Intuitive Surgical Inc, Sunnyvale, CA) became the first assisting surgical robot to receive FDA approval to assist surgeons in performing laparoscopic surgery [3]. The use of this novel technology has enabled surgeons to overcome the limitations of more traditional laparoscopic and thoracoscopic surgeries. Among its advantages are improved dexterity and increased degree of freedom, 3D visualization allowing improved hand-eye coordination, and position reducing fatigue [4]. It has also been shown to reduce intraoperative blood loss, intraoperative and postoperative complications, and, most importantly, improve patient's quality of life [5,6].

Robotic surgery is rapidly spreading across various specialties, with an annual increase of around 15%. In 2020, its global volume was 1.24 million, with the United States (US) accounting for 70.6% [7]. Every year, more than 900 new robotic platforms are installed around the world [7]. In England, 48 of 149 (32% of acute NHS trusts) had at least one surgical robot [8]. In 2018, these robotic centers performed nearly 12,000 robotic surgeries, 83.4% of which were urological procedures [8]. However, data on the use of robotic surgery in low- and middle-income countries is limited (LMICs). The incorporation of robotic surgery into LMIC healthcare systems has the potential to be beneficial. This review discusses the advantages, disadvantages, potential improvements, and the future trajectory of robotic surgery in LMICs.

## 2 Recent robotic surgery advancements and applications

Robotic surgery has been widely incorporated into common practice in the 21st century. There are nearly 70 representative clinical uses for da Vinci systems, spanning clinical specialties, including urology, gynecology, thoracic surgery, general surgery, and transoral surgery [4,7]. The FDA has cleared robotically-assisted surgical (RAS) devices for use by trained physicians in an operating room environment for laparoscopic surgical procedures in general surgery, cardiac, colorectal, gynecologic, head and neck, thoracic and urologic surgical procedures [9].

Robotic-assisted surgery has quickly expanded in the field of urology, and one of the most established procedures is robot-assisted prostatectomy, with a robotic utilization rate of around 80% in the United States [10,11]. Other robot-assisted urologic procedures include radical cystectomy, nephrectomy, pyelolithotomy, nephrolithotomy, distal ureteric reconstruction, retroperitoneal lymph node dissection, augmentation enterocystoplasty, and artificial urinary sphincter insertion [12].

The role of robotic surgery is well-established in benign gynecological procedures, including robotic myomectomy, robotic hysterectomy, robotic endometriosis eradication, and robotic pelvic organ prolapse treatment [13]. In 2010, over 1200 gynecologic surgeons were trained to use the da Vinci robotic system [14]. In the US, the robotic volume for definitive oncologic resection in the uterus and cervix was 44.9% and 36.9% respectively [11].

In general surgery, the use of robotic surgery increased significantly from 1.8% in 2012 to 15.1% in 2018 [13]. The number of surgeons who performed robotic general surgery also increased from 8.7% in 2012 to 35.1% in 2018 [13]. Common general surgery procedures performed using robotic surgery includes inguinal hernia repair (28.8%), proctectomy (26.7%), reflux surgery (26%), ventral hernia repair (22.4%), colectomy (16.3%) and cholecystectomy (7.5%) [13]. The robotic utilization in colorectal procedures in the US was 7.5% in 2015 [10]. Robotic surgery has also gained popularity in the field of general surgical oncology. In 2010, only 1% of the surgical oncology operations were robotic, but by 2014, the percentage of robotic cases increased to 6%, achieving a 5-fold increase [14].

Robotic surgery was also used in pediatric surgery, where the most common application is in urology [15]. The use of robotic surgery was also reported in other pediatric surgical subspecialties, such as otolaryngology, general surgery, thoracic surgery, and surgical oncology, but it is often limited by the availability of appropriately sized instruments for the pediatric body habitus [15].

## 3 Newer robot-assisted surgery techniques: applications, benefits, and limitations

i. Robotic Endoscopic Catheterization

Due to the disadvantages of traditional vascular interventional surgery (VIS), such as surgical risks and complications, and exposure to prolonged radiation from imaging, robotic interventions, specifically robot-assisted endovascular catheterization (EC) systems, are gaining attention. These techniques may also enable remote telesurgery, particularly in medically underserved areas such as rural areas [16]. Furthermore, traditional catheterization employs ultrasound to visualize the soft tissue and catheter to ensure proper placement and fluoroscopy to visualize the catheter, with the latter exposing the patient and physician to radiation and the former producing noisy images with limited resolution. To improve the operating experience for both the patient and the physician, robotic EC systems that use haptic force feedback, machine learning, and image processing algorithms may be used [17].

Transcatheter closure has traditionally been the preferred method for treating atrial septal defect (ASD) [18]. Robotic endoscopic surgery, on the other hand, has become the least invasive method of ASD repair. A retrospective analysis revealed that of the 462 patients who underwent ASD closure at a Turkish hospital, 217 underwent totally endoscopic robotic surgery and 245 underwent transcatheter closure [18]. The length of ICU, and hospital stay, was significantly longer for patients undergoing ASD repair via robotic surgery; however, postoperative complications were minimal and comparable in both groups, indicating that both procedures are relatively low risk [18]. Because of the minimal invasiveness of the procedure, robotic surgery has a comparable complication risk and may be superior for cosmetic advantage and patient comfort.

ii. Robot-assisted Invasive Heart Surgery: Valve Replacement

In the treatment of coronary artery disease (CAD), hybrid coronary revascularization has become more common. Hybrid revascularization is commonly used in minimally invasive coronary artery bypass grafting (CABG) and percutaneous coronary intervention procedures. A study conducted in China found that none of the patients who underwent robotic-assisted left internal mammary artery harvesting experienced postoperative complications [19]. Even though conventional CABG still accounts for the vast majority of CABG operations, robotic-assisted surgeries are becoming more common. Between 2012 and 2017, 1,204,125 adults in the United States underwent non-robotic CABG and 7355 underwent robotic CABG [20]. The robotic CABG group had a lower risk of in-hospital mortality, acute kidney injury, post-operative hemorrhage, and lower transfusion, cost, and length of hospital stay. However, the rates of stroke and sepsis in both groups were comparable [20]. This data suggests that robotic-assisted CABG may be an effective method for reducing some post-operative complications while also reducing the burden on patients and their families by reducing cost and hospital stay length.

While total endoscopic coronary artery bypass (TECAB) is typically used on left coronary arteries, it has recently been used for right coronary artery (RCA) grafting. RCA access is typically difficult due to anatomic and technical challenges, but a study has suggested that robotic beating-heart TECAB in RCA disease is a feasible and useful option [21]. Robotic surgical machines have numerous potential applications in invasive heart surgery. More research is needed to determine the absolute effectiveness of robotic devices in high-risk surgeries, but there is promising evidence for the use of robotic devices to reduce patient burden, postoperative complications with a less invasive procedure, and provide care to medically underserved areas.

ii i.Robot assisted Head and Neck Surgery

In head and neck surgery, the majority of pharyngeal procedures are now performed using transoral robotic surgery with the DaVinci system [22]. Several studies have shown improved post-operative injury outcomes, quality of life, and increased swallowing function preservation [23]. Transoral robotic surgery with a thulium YAG laser has been shown to improve larynx surgery significantly when compared to traditional electrocautery surgery [24]. The implications are especially beneficial for larynx surgery, which requires specialized minuscule instruments [23]. Transoral robotic nasopharynx surgery has shown that using the DaVinci surgical device can potentially eliminate the need for the “palatine split” normally associated with general nasopharynx surgery [25]. This enables visualization of anatomical landmarks within the cavity using a method that reduces morbidity associated with the “palatine split” [23]. In terms of the sinus and anterior skull, robotic surgery extends to the medial orbit, ethmoid sinuses, cribriform plate, sella and surrounding structures, pterygopalatine fossa, and clivus [23]. This procedure allows for examination of structures such as cranial nerves IX-XII, internal and external carotid arteries, and the jugular vein as they pass through the anterior lateral skull [23]. In cadaver models, robotic-assisted surgery has been shown to be beneficial in achieving more precise cochlear implants as well as delivering more precise mastoidectomies [23]. Transoral robotic neck dissection via a gasless postauricular facelift approach is an excellent procedure for improving postoperative outcomes and cosmetic features [26]. This procedure's applicability to other invasive neck procedures, such as oncological procedures, should be investigated [26].

## 4 Potential benefits of robotic surgery integration in LMICs

Aside from the widely discussed general benefits of robotic surgery, its integration has significant potential benefits in LMICs. Reducing surgical site infection is critical for ensuring access to safe surgical care [27], and robotic surgery could be a solution. Several studies have found that LMICs have a higher incidence of surgical site infection (SSI) and a higher rate of antibiotic resistance SSI [27,28]. In LMICs, SSI may result in a longer hospital stay, delaying patients' return to school or work and lowering overall productivity [27]. In robotic surgery, surgical incisions are smaller than in open surgery, lowering the risk of SSI, indirectly reducing antibiotic use, and preventing the development of antibiotic resistance.

Furthermore, robotic surgery allows surgeons and staff to be physically separated from the patient, reducing the risk of infectious disease transmission [29]. This is especially beneficial in LMICs during and after the COVID-19 era. Patients could also be discharged sooner after surgery [29], lowering the risk of nosocomial infection and freeing up hospital beds. Tertiary hospitals in low-income countries are overcrowded, with some exceeding capacity by 200–300% [30]. As a result, in LMICs with limited healthcare resources, a shorter hospital stay is critical for optimizing surgical service delivery.

LMICs are home to nearly half of the world's population, but only 19% of all surgeons [31]. While efforts should be directed toward increasing the surgical workforce, robotic surgery may play a role. Telementoring and telesurgery may be possible with the integration of robotic surgery. Telesurgery allows surgeons to operate remotely, reducing travel time and eliminating geographical barriers, resulting in increased surgical output [3,32]. Telesurgery also enables experienced surgeons to demonstrate operative steps to less-experienced surgeons and may even provide real-time guidance to operating surgeons, improving surgical outcomes [3,32].

The incorporation of robotic surgery into the healthcare system may aid in the reduction of global surgical disparities. As previously stated, robotic surgery is a developing field that has been extensively researched and integrated into practice. Early robotic surgery application provides educational and research opportunities in LMICs, reducing inequity in surgical access and delivery. Institutions that have robotic surgical systems may be able to serve as local hubs for robotic surgery education, research, and clinical support to other centers across the country. Researchers in low- and middle-income countries (LMICs) could innovate or transform the robotic surgical system and equipment to better suit local healthcare settings. To improve cost-effectiveness, LMICs could also manufacture their own robotic surgical instruments. This could help to close the gap in surgical access between low- and middle-income countries (HICs).

Depending on the type of surgery, robotic surgery could potentially reduce the cost of healthcare services. The authors discovered that the total cumulative cost of robotic-assisted radical prostatectomy and open radical prostatectomy is similar one year after discharge in a retrospective cohort study [33]. Even though the cost of robotic-assisted radical prostatectomy was higher than that of open radical prostatectomy during the index hospitalization, the postdischarge health care use was significantly lower, which offset the initial cost [33].

## 5 Potential challenges to robotics surgery integration in LMICs

Despite its promise, there is a huge disparity in access to robotic surgery. HICs have spearheaded innovative developments in robotic techniques that would assist in the precision of surgery. However, these innovations have not yet reached low-income countries due to the lack of financial infrastructure. Implementing a new robotic surgical platform possibly costs over 1 million USD and an additional 3,000–5,000 USD per surgical procedure [34,35]. Worth considering are prevalent financial constraints on patients related to transportation, and lack of insurance coverage, especially in middle-income countries, as a study conducted in Columbia calculated robotic cardiac surgery to cost extra in ASD repairs and mitral valve repairs at 2,044 USD and 2,200 USD respectively, compared to traditional procedures [[36], [37], [38]]. Robotic surgery was feasible in the long term due to its shorter stay, however, immediate costs remain high [39]. Furthermore, there is a scarcity of data detailing costs of robotic platforms, and maintenance which makes it difficult to ascertain [39]. Underserved communities and LMICs are disproportionately affected as robotic surgery remains accessible to only wealthy communities, further perpetuating the vicious cycle of socioeconomic inequity. [40].

Shortage of surgeons, lack of training, as well as network issues, also contributes to the inequitable community-wide implementation of robotic surgery. Training is not standardized in surgical practice, thereby increasing the risk of medical errors and jeopardizing patient safety in countries that do not provide general training [31]. Simulation training by utilizing 3D models may help but these novel technologies may not reach underserved communities [41]. Recent advances such as remote telesurgery can possibly help bridge the robotic surgery gap in remote areas. However, it is estimated that a delay of 300 ms was the maximum delay that is compatible with safe robotic surgery and can become compromised in areas with poor network connectivity [42]. Though 5G internet technology and ATM fibers can reduce the delay, their implementation may take another 3–5 years in low-income countries [35,43].

## 6 Recommendations and conclusion

One of the primary factors limiting access is socioeconomic constraints. Universal robotic technology licensing can boost competitiveness and product availability, potentially resulting in lower installation costs. Other potential approaches to reducing the load include encouraging HICS to share resources and equipment, establishing a national cloud system sponsored by various nations, and establishing subsidies to allow for financial assistance in implementation for hospitals in more remote locations. This, in addition to implementing subsidies for hospitals in more remote areas, can help to alleviate the strain. Furthermore, investments in transportation and logistics may aid in improving access to remote areas.

Proper use of robots in surgery necessitates the acquisition of training, which may include dry and wet lab practice, modular console training, and independent practice, which is especially difficult to obtain in LMICs. Twinning programs, in which a HIC institution collaborates with an LMIC facility to increase access, training, and research opportunities, aiding in the expansion of robotic training options.

Overall, robotic surgery has a lot of potential benefits for LMICs.More research addressing regional challenges and improvements is crucial to wider implementation of robotic surgery to improve surgical care in LMICs.

## Ethical approval

N/A.

## Sources of funding

N/A.

## Author contribution statement

Substantial contribution to the Conception and design of the work: All authors under the guidance of Aashna Mehta and Wireko Andrew Awuah. Drafting the work and critical revision: All authors under the guidance of Arda Isik, Aashna Mehta, Wireko Andrew Awuah, Mohammad Mehedi Hasan, and Vladyslav Sikora. All the authors read and approved the final version of the manuscript.

## Registration of research studies

Name of the registry: N/A.

Unique Identifying number or registration ID: N/A.

Hyperlink to your specific registration (must be publicly accessible and will be checked): N/A.

## Guarantor

Mohammad Mehedi Hasan.

Department of Biochemistry and Molecular Biology, Faculty of Life Science, Mawlana Bhashani Science and Technology University, Tangail, Bangladesh; mehedi.bmb.mbstu@gmail.com (MMH).

## Consent

N/A.

## Funding statement

Authors have no funding to declare.

## Data availability statement

No data available.

## Declaration of competing interest

Authors have no conflicts of interest to declare.
